# A Synbiotic of *Bifidobacterium animalis* subsp. *lactis* BB-12 and 2′-FL Alleviate Infant Diarrhea and Anxiety-like Behaviors via Gut Microbiota Modulation in an EPEC O127 Infection Model

**DOI:** 10.3390/nu17193099

**Published:** 2025-09-29

**Authors:** Zhuo Liu, Wenxiu Wang, Ning Li, Jinkuan Chen, Qianxu Wang, Mengzhen Jia, Xiaorui Wang, Bo Zhang, Nan Sheng, Zhigang Liu

**Affiliations:** 1 College of Food Science and Engineering, Northwest A&F University, Yangling 712100, China; liuzhuo@jlbry.com (Z.L.); wangwenxiu@nwafu.edu.cn (W.W.); chenjinkuan2022@nwafu.edu.cn (J.C.); wangqianxu@nwafu.edu.cn (Q.W.); jiamengzhen@nwafu.edu.cn (M.J.); wangxiaorui0105@nwafu.edu.cn (X.W.); 18864606056@nwafu.edu.cn (B.Z.); 2 Research and Development Center, Xi’an Yinqiao Dairy (Group) Co., Ltd., Xi’an 710075, China; 18392059055@163.com; 3 Nutrition Research Institute, Junlebao Dairy Group Co., Ltd., 36 Shitong Road, Shijiazhuang 050221, China; shengnan@jlbry.com; 4 Shenzhen Research Institute, Northwest A&F University, Shenzhen 518000, China

**Keywords:** infant diarrhea, bifidobacterium, synbiotic, 2′-fucosyllactose, gut microbiota, SCFAs, escherichia, Akkermansia

## Abstract

**Background/Objectives**: Infant diarrhea is a major global cause of morbidity and mortality. While *Bifidobacterium* is linked to diarrhea, its preventive effects, underlying mechanisms, and potential synergistic benefits with prebiotics remain unclear. The objective of this study was to explore the efficacy of a synbiotic composed of *Bifidobacterium animalis* subsp. *lactis* BB-12 (BB-12) and 2′-fucosyllactose (2′-FL) in alleviating infant diarrhea. **Methods**: One-week-old C57BL/6J mice were used to construct a model of infant diarrhea via infection with enteropathogenic *Escherichia coli* (EPEC) O127. Mice were administered BB-12 (10^8^ CFU per mouse), 2′-FL (1 g/kg), or their combination (synbiotic) for three consecutive weeks. **Results**: Administration of the synbiotic not only markedly improved diarrhea, anxiety-like behavior, colon inflammation, and gut barrier function but also positively reshaped the microbial community. This was achieved through a significant rise in short-chain fatty acid (SCFA)-producing bacteria (e.g., *Akkermansia* and *Paraprevotella*), a rise in fecal SCFAs, and a reduction in harmful bacteria such as *Escherichia*. **Conclusions**: The synbiotic effectively relieves EPEC-induced infant diarrhea by regulating gut microbiota composition and metabolic functions. These findings highlight its potential as a dietary intervention in infant diarrhea and provide new insights into infant health applications.

## 1. Introduction

Among infant populations worldwide, infectious diarrhea continues to pose a major threat to health and survival, particularly in developing nations, posing a significant threat to child health and development [1]. This condition, often accompanied by anorexia and fever, impairs nutrient absorption and may result in serious complications including dehydration and multi-organ failure [2,3]. As a key pathogenic serotype, Enteropathogenic *Escherichia coli* (EPEC) has been established as a main etiological agent responsible for moderate to severe diarrhea in infants [4,5]. Studies have shown that EPEC has the potential to lead to the impairment of gut barrier function and the dysbiosis of gut microbiota [6]. The gut barrier’s first line of defense is a mucus layer, principally formed by mucin-2 (MUC2) secreted from goblet cells, which physically prevents pathogen access to epithelial cells [7,8]. EPEC infection compromises this layer and further damages epithelial integrity by downregulating key tight-junction proteins like Claudin-1, leading to increased inflammation, gut permeability, and gut microbiota dysbiosis [9,10].

Given that the gut ecosystem plays a central role, microbiota-targeted interventions are a promising therapeutic strategy [11]. Probiotics, such as *Bifidobacterium animalis* subsp. *lactis* BB-12, have demonstrated clinical efficacy in reducing the risk and duration of infant diarrhea [12]. Research shows that BB-12 can impede pathogens via the synthesis of antibacterial substances and competing for attachment to the mucosal surface [13]. An in vitro study has shown that BB-12 can increase the strength of tight junctions and prevent impairment to the functional integrity of the epithelial barrier [14]. BB-12 alleviated gut injury in mice with acute pancreatitis and altered the gut microbiota composition [15]. Clinical evidence further shows that BB-12 can reduce the risk that affects infants and young individuals and decrease the average number of diarrhea days by up to 50% [16]. Moreover, in instances of antibiotic-associated diarrhea, BB12 accelerates the recovery of SCFAs and reduces the incidence of diarrhea [17]. However, single-strain probiotics such as BB-12 still face limitations, including gastric acid sensitivity, dose-dependent efficacy, and functional specificity. A synbiotic strategy can optimize the application efficacy of BB-12.

Human milk oligosaccharides (HMOs), with 2′-fucosyllactose (2′-FL) being a typical instance, represent ideal prebiotics. They are indigestible by the host and act as selective fuel for beneficial bacteria, especially *Bifidobacterium* [18]. Furthermore, one study had shown that 2′-FL can independently inhibit *Escherichia coli* (*E. coli*) adhesion, enhance gut barrier function, and increase the production of SCFAs—key microbial metabolites that regulate gut health and inflammation [8]. The combination of BB-12 and 2′-FL is particularly compelling, as BB-12 possesses the specific enzymatic machinery to efficiently utilize 2′-FL, a trait not universal among probiotics [19]. This selective advantage suggests a powerful synergistic potential, yet it has not been tested in the context of EPEC-induced infant diarrhea.

To evaluate the therapeutic potential of a synbiotic containing BB12 and 2′-FL against infant diarrhea, an EPEC-induced model was developed in 1-week-old C57BL/6J mice. Following pathogen exposure, the animals received three-week interventions comprising BB12, 2′-FL, or their combinations. Comprehensive evaluations included longitudinal monitoring of diarrheal severity, behavioral assessments for anxiety-like behaviors, quantification of pro-inflammatory factors, immunohistochemical analysis of gut barrier markers (MUC2 and Claudin-1), 16S rRNA sequencing for microbial profiling, and gas chromatography-mass spectrometry for fecal SCFAs quantification. Multivariate correlation analyses were subsequently conducted to establish interconnections among microbial taxa, metabolic signatures, and behavioral phenotypes. These findings advance our understanding of microbiota-targeted nutritional strategies for diarrheal management, highlighting the translational promise of synbiotic formulations in pediatric gastroenterology.

## 2. Materials and Methods

### 2.1. Chemicals

All chemicals and reagents were sourced from reliable suppliers and selected for their high purity. Among them, HMOs were purchased from DSM-Firmenich. The commercial strains of *Bifidobacterium animalis* subsp. *lactis* BB12, *Lactobacillus acidophilus* NCFM, *Lactobacillus paracasei* L9300BH, *Bifidobacterium animalis* subsp. *lactis* Bi-07, *Bifidobacterium animalis* subsp. *lactis* HN019, *Lacticaseibacillus rhamnosus* HN001, and *Lacticaseibacillus rhamnosus* MP108 were provided by Beijing HeyiYuan Biotechnology Co., Ltd. Primary antibodies were obtained from China Abcam (Shanghai, China). Secondary antibodies and associated reagents were acquired from Servicebio (Wuhan, China). Meanwhile, the kit was sourced from Solarbio Science & Technology (Beijing, China).

### 2.2. Probiotic Screening Based on 2′-FL

Transfer the seven strains that are allowed to be added to infant formula food to de Man, Rogosa and Sharpe medium (MRS) liquid culture medium and culture them at 37 °C for at least 24 h. Inoculate the strains separately in MRS liquid medium and liquid medium with 2′-FL as the dominant carbon source. The growth curves of the strains in different carbon source media were measured by a fully automatic microbial production curve analyzer, and 1 strain of probiotics that can utilize 2′-FL was screened to determine the composition of the symbiotic elements. The specific operation is as follows: Take the cultured bacterial solution for later use and prepare culture media containing different carbon sources. Add the bacterial solution and culture medium in a ratio of 50 μL to 300 μL to a 100-well plate in an anaerobic operating station. Seal the 100-well plate with a sealing film to maintain the anaerobic environment, and then place it in a fully automatic growth curve analyzer. Plot the growth curve of microorganisms by measuring the OD600 value change in small holes for 24 h.

### 2.3. Animal Experimental Design

Wild-type CBSBL/6J mice (6–8 weeks old) were obtained from Jicui Yaokang Bio-technology Co., Ltd. (Nanjing, China). All animals were maintained under controlled conditions (temperature: 22 ± 2 °C; humidity: 50 ± 5%; 12 h/12 h light/dark cycle) with free access to food and water. After a 7-day acclimation period, female and male mice were paired at a 2:1 ratio for mating. On postnatal day 7, the offspring were randomly divided into five experimental groups (*n* = 8 per group), with a total of 40 mice used in the study. The sample size of mice in this study was determined mainly with reference to two previously published peer-reviewed studies in the same field [20,21]. These studies have verified the reliability and statistical validity of results under a specific sample size for mouse experiments using EPEC-related infection models. Based on this, we calibrated the sample size parameters in combination with the primary outcome measure of ‘diarrhea incidence’ in our study. During the calculation, the statistical power (1-β) was set to 0.85 and the confidence level (1-α) was set to 0.95. Finally, the minimum required sample size per group was estimated to be 8 mice.

Administration of EPEC O127:K63:H6 (1 × 10^10^ CFU per mouse) via daily oral gavage established the diarrhea model [21]. The CON group received an equal volume of PBS. Following the EPEC challenge, the treatment groups were administered either 2′-FL (1.0 g/kg) or BB12 (10^8^ CFU) bacterial suspension, both delivered in a volume of 100 μL. The control group received PBS at 10 μL/g body weight. The body weight of mice was documented at three-day intervals. Before weaning on day 21, pups were breastfed; thereafter, a maintenance diet (TROPHIC Animal Feed High-Tech Co., Ltd., Nantong, China) was provided. At four weeks of age, fecal samples were collected and behavioral tests were conducted. Subsequently, mice were euthanized for tissue and serum collection, and all samples were stored at −80 °C to be used for subsequent analysis. The study included five groups: CON (control), EPEC (diarrhea model), EPEC + 2′-FL, EPEC + BB12, and EPEC + 2′-FL + BB12.

All animal experiments were carried out in line with the Guide for the Care and Use of Laboratory Animals (8th edition, ISBN-10: 0-309-15396-4). The study was reviewed and approved by the Animal Ethics Committee of Northwest A&F University, with the following details: Ethic Committee Name: Animal Ethics Committee of Northwest A&F University. Approval Code: XN2023-0710. Approval Date: 22 May 2023.

### 2.4. Bacterial Cultivation

EPEC O127:K63:H6 was recovered by culturing in Luria–Bertani (LB) broth at 37 °C for at least 24 h. The culture was then subcultured into fresh LB medium and incubated again under the same conditions for another 24 h. Bacterial cells were collected by centrifugation, washed with PBS, and centrifuged again to remove the supernatant. The pellet was resuspended in 1 × PBS, and the bacterial concentration was adjusted to approximately 10^11^ CFU/mL based on plate counting. Similarly, 7 strains of probiotics were initially activated in MRS broth at 37 °C for no less than 24 h, followed by subculturing in fresh MRS under identical conditions. After incubation, cells were harvested by centrifugation, washed with PBS, and recentrifuged. The final pellet was resuspended in 1 × PBS and diluted to approximately 10^9^ CFU/mL as quantified by plate counting.

### 2.5. Animal Behavioral Assessment

#### 2.5.1. Open Field Test

Before sacrifice, mice were subjected to an open field test to measure anxiety-like behavior [22]. The behavioral test was conducted in a square plastic arena (40 cm × 40 cm × 40 cm), which was divided into a central area (24 cm × 24 cm) and a peripheral zone. At the beginning of each test session, each mouse was positned gently in the center of the arena and permitted to explore freely for 5 min. To eliminate interference from residual odors between trials, the apparatus was meticulously wiped with 75% ethanol after each test. A video tracking system (SuperMaze) was used to automatically record and analyze two key indicators: the distance moved in the central zone (reflecting anxiety-related avoidance of novel open spaces—anxious mice typically show less movement in the unprotected central area) and the total locomotion distance (to exclude the influence of overall locomotor activity on anxiety assessment, ensuring differences in central zone distance are not due to reduced mobility).

#### 2.5.2. Elevated Cross Maze Test

The elevated cross maze (consistent with the internationally recognized elevated plus maze, EPM, a classic method for measuring anxiety responses) was used to further evaluate anxiety-like behaviors. The apparatus consists of two open arms (30 cm long × 8 cm wide), two closed arms (30 cm long × 8 cm wide), and a central area (8 cm × 8 cm), with the entire maze elevated 70 cm above the ground. During the test, each mouse was placed in the central area, facing an open arm, and its behavior was recorded for 5 min using a video tracking system (SuperMaze). The recorded parameters focused on distance-related indicators, including the distance moved in the open arms, the distance moved in the closed arms, and the total locomotion distance in the entire maze. Subsequently, two core anxiety-related indicators were calculated based on these distance data: the proportion of open arm movement distance (percentage of total maze movement distance accounted for by distance in open arms).

#### 2.5.3. Gastrointestinal Transit Time (GITT)

GITT was evaluated in mice before euthanasia. A 6% (*w*/*v*) carmine red solution was formulated by dissolving the dye in 0.5% methylcellulose, which served as a non-absorbable vehicle. Oral gavage was used to deliver 0.3 mL of this solution to each mouse, and the moment of administration was set as T_0_. Throughout the procedure, animals had free access to food and water and were able to move freely to avoid stress-induced artifacts. The time at which the first red fecal pellet was observed was recorded as T_end. GITT was calculated as the difference between T_end and T_0_.

#### 2.5.4. Diarrhea Score

Diarrhea scores were assessed weekly in mice starting from the second week of age [6]. Fecal consistency was scored on a 0–4 scale as follows: 0, normal, well-formed pellets; 1, pasty stools adhering to the cage wall; 2, loose stools with possible mucus; 3, watery stools with possible mucus; 4, bloody stools.

### 2.6. Collection of Mouse Feces, Blood Samples, and Specimens

As previously described [23], following the three-week intervention period, fecal samples were collected by individually placing mice into sterilized 500 mL beakers labeled with their respective IDs. Fresh fecal pellets were quickly transferred to 1.5 mL sterile centrifuge tubes, snap-frozen with liquid nitrogen, and kept at −80 °C for subsequent *t*-tests.

Before sacrifice, all mice were fasted for 12 h, with free access to drinking water during this period. After fasting, the mice were anesthetized via intraperitoneal injection of 1.25% (*v*/*v*) sterile tribromoethanol, with the dose set at 0.2 mL per 10 g of body weight. A stable anesthetic state was confirmed by the loss of the mice’s righting reflex and paw withdrawal reflex before proceeding to the next step. Under stable anesthesia, blood was collected from the mice through eyeball enucleation. The collected blood was centrifuged at 2500 rpm for 15 min at room temperature to separate and obtain serum, which was then stored at −80 °C. After blood collection, the mice were euthanized using the cervical dislocation method. Finally, tissues including the gut and other visceral organs were collected for subsequent experimental analysis.

### 2.7. Hematoxylin and Eosin (H&E) Staining

As detailed previously [24], subsequent to euthanasia, colon tissues were immediately collected and placed in 4% (*v*/*v*) paraformaldehyde (PFA)/PBS fixative for 24 h of fixation. Once dehydrated, the tissues were embedded in paraffin, followed by sectioning into 5-μm-thick slices using a microtome. Sections were floated on a 42 °C water bath to flatten and mounted onto APES-coated slides. Slides were dried in a 37 °C constant-temperature oven. For dewaxing and rehydration, tissue sections were sequentially immersed in xylene I (10 min), xylene II (10 min), 100% ethanol I (5 min), 100% ethanol II (5 min), 90% ethanol (5 min), 80% ethanol (5 min), and 70% ethanol (5 min). Sections were then washed three times with PBS (5 min each). Nuclear staining was performed with hematoxylin for 5 min, followed by differentiation in 1% hydrochloric acid-ethanol, rinsing under running water for 15 min, and bluing in 1% dilute ammonia water solution. Cytoplasmic staining was achieved with eosin for 3 min. Sections underwent conventional ethanol dehydration, xylene clearing, and final mounting with neutral resin to prevent air bubbles. After air-drying, sections were observed and photographed under an optical microscope.

### 2.8. Alcian Blue Staining

According to the instructions of the kit manufacturer, use the Alcian Blue staining kit to stain the tissue sections. Place the sections in an oven at 37 °C overnight to remove moisture. Dewax the sections with xylene for 5 min, rehydrate them with gradient alcohol for 5 min, and then hydrate them with distilled water for 5 min. Immerse the sections in an acidic solution for 3 min, and then gently shake off the liquid from the sections. Stain the sections with the staining solution for 20 min, and rinse them with running water for 5 min. Then counterstain the sections with nuclear fast red staining solution for 10 min, and rinse them with running water for 1 min. Dehydrate the sections with gradient ethanol for 10 s, carry out transparent treatment with xylene for 1 min, seal the sections with neutral balsam, let them air dry, and then observe the colonic tissue sections under an optical microscope. Use the ImageJ software (64-bit Java 8) to conduct a quantitative analysis of the Alcian Blue-stained goblet cells.

### 2.9. Immunofluorescence Staining

Immunofluorescence staining was conducted on colon tissue sections to assess the expression of MUC2 and Claudin-1, following a previously reported protocol [25]. The specific experimental steps were as follows: Dry tissue sections at 37 °C overnight; after deparaffinization with xylene and rehydration via ethanol gradient, wash 5× with PBS (4 min each). Permeabilize sections with permeabilization solution at room temp for 15 min, wash 6× with PBS (3 min each); perform heat-induced antigen retrieval, then wash 6× with PBS (5 min each) and blot excess liquid. Block sections with goat serum at 37 °C for 10 min; add primary antibody and incubate at 4 °C overnight. After 8 PBS washes, add fluorescent secondary antibody, incubate at 37 °C for 2 h; wash 8× more with PBS and mount with DAPI-containing anti-fade medium. Capture images via inverted fluorescence microscope; quantify MUC2 and Claudin-1 expression using ImageJ.

### 2.10. Enzyme-Linked Immunosorbent Assay (ELISA)

According to the manufacturer’s instructions, measure the levels of inflammatory cytokine TNF-α and anti-inflammatory cytokine IL-10 in the colon using an ELISA kit. Weigh 50 mg of colon tissue and vortex homogenize it in 500 μL of 1 × PBS. Centrifuge the supernatant and set it aside for later use. Establish a standard curve and use it to calculate the level of indicators in the sample.

### 2.11. qRT-PCR Analysis

Total RNA was isolated from colon tissue using BIOZOL reagent (Hangzhou Bioer Technology Co., Ltd., Hangzhou, China), following a previously described method [26]. RNA concentration was determined with a NanoDrop 2000/2000C spectrophotometer (Thermo Fisher Scientific, Waltham, MA, USA), and all RNA samples were adjusted to a uniform concentration using nuclease-free water.

Subsequently, cDNA was synthesized using the UEIris RT Mix with DNase (Suzhou Hengyu Biotechnology Co., Ltd., Suzhou, China). The synthesized cDNA was diluted fivefold in nuclease-free water, and quantitative real-time PCR (qPCR) analysis was then performed using 2× SYBR Green qPCR Master Mix (provided by the same manufacturer as the RT mix).

GAPDH served as the endogenous reference gene for normalization, and the relative expression levels of target genes were calculated using the 2^−ΔΔCt^ method. The nucleotide sequences of all primers employed in this experiment are listed in Table A1.

### 2.12. 16S rDNA Amplicon Sequencing

DNA quality was confirmed via 1% agarose gel electrophoresis, while its concentration and purity were assessed using spectrophotometry. PCR amplification was performed on the V3–V4 hypervariable regions of bacterial 16S rDNA with region-specific primers, and amplicon quantities were measured using a QuantiFluor ST fluorometer (Promega Corporation, Madison, WI, USA). Equimolar volumes of PCR products were combined to generate a sequencing library.

Sequencing was carried out on the Illumina MiSeq platform, with raw data processed through the BGI Genomics Analysis Platform. Bioinformatic and statistical analyses were subsequently conducted using R-based packages and relevant tools. Alpha diversity, represented by Chao1 and Simpson indices, was computed to evaluate microbial richness and evenness within samples.

Community structural similarities were visualized via Venn diagrams and principal coordinate analysis (PCoA) based on OTU clustering. LEfSe analysis was used to identify differentially abundant microbial taxa and potential functional characteristics across various taxonomic levels.

### 2.13. Fecal SCFAs Level Analyses

SCFAs quantification was performed via gas chromatography (GC), following a previously established protocol [27]. A standard curve was constructed using serial dilutions of a mixed SCFAs standard solution. For sample preparation, 0.15–0.20 g feces in 1 mL ultrapure water + 0.15 mL 50% (*w*/*w*) sulfuric acid; add 1.6 mL diethyl ether, incubate, centrifuge (8000 rpm, 5 min); collect supernatant, filter (0.2 µm membrane, Branch Billion Lung, Tianjin, China), and transfer to vials for GC.

Acetate (cat. A116173), propionate (cat. P110445), butyrate (cat. B11se0438) standards were from Aladdin Bio-Chem (Shanghai, China). GC separation used a Shimadzu GC-2014C (Kyoto, Japan) with an Agilent DB-FFAP capillary column (Santa Clara, CA, USA) and flame ionization detector.

### 2.14. Data Processing and Analysis

All data represent the mean ± SEM of at least three independent experiments and were analyzed for statistical significance using one-way ANOVA with Tukey’s post hoc test (GraphPad Prism 6.0). Associations between variables were assessed using Spearman’s correlation and principal coordinate analysis (PCoA), performed in RStudio (v3.4.1, Boston, MA, USA). A *p*-value below 0.05 was considered statistically significant.

## 3. Results

### 3.1. Selection of Optimal Probiotics with 2′-FL Serving as the Sole Carbon Source

**Figure 1:**
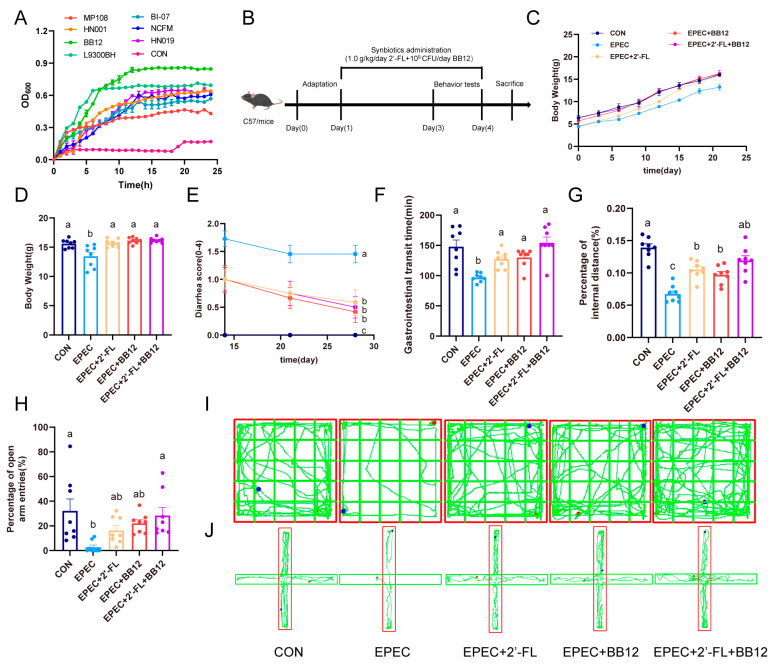
Impact of synbiotic supplementation on EPEC-induced infant diarrhea and anxiety-like behaviors. (**A**) the growth curve at OD600; (**B**) experimental timeline for animal treatments; (**C**) body weight; (**D**) body weight at the fourth week; (**E**) diarrhea scores (0–4); (**F**) GITT; (**G**) open field test; (**H**) elevated cross maze test; (**I**) open field test trajectory map; (**J**) elevated cross maze test trajectory map. Data are expressed as mean ± SEM, *n* = 8. The letters (a–c) denote significant differences (*p* < 0.05) among the treatment groups.

In the culture medium with prebiotic 2′-FL in the role of the only carbon source, the OD600 value of BB-12 is the highest at 24 h, and the rate of reaching the growth stationary phase is relatively fast (Figure 1A). Therefore, BB-12 was selected and combined with 2′-FL to form a synbiotic to study the effects of the synbiotic on EPEC-induced infant diarrhea.

### 3.2. Impact of Synbiotic Supplementation on EPEC-Induced Infant Diarrhea and Anxiety-like Behaviors

For the purpose of examining the influence of probiotics, prebiotics, and synbiotics on diarrhea symptoms and anxiety-like behaviors in a mouse model of EPEC-induced diarrhea, one-week-old C57BL/6 mice were allocated to five experimental groups. Diarrhea was induced through daily oral administration of EPEC O127:K63:H6 at a dose of 10^10^ CFU per mouse. After weaning, the mice underwent a three-week intervention with their respective treatments. Upon completion of the intervention, all animals were euthanized to assess the effects of HMO-based treatments on diarrhea-associated symptoms (Figure 1B). During the majority of the experiment, body weight did not vary significantly across the five groups (*p* > 0.05; Figure 1C), by the fourth week, the EPEC-infected group exhibited a 13.7% reduction in body weight compared to the CON group (*p* < 0.01). Supplementation with probiotics, prebiotics, or synbiotics significantly mitigated this EPEC-induced body weight loss (*p* < 0.01; Figure 1D).

Diarrhea severity was assessed weekly beginning once the mice reached two weeks of age. The EPEC-infected group showed elevated diarrhea scores, which were significantly reduced by synbiotic treatment (*p* < 0.01; Figure 1E). GITT was also measured as an indicator of gut motility and diarrhea symptoms. EPEC infection shortened GITT, but this effect was reversed by synbiotic administration (*p* < 0.01); probiotics and prebiotics also improved GITT, albeit to a lesser extent (*p* < 0.05; Figure 1F).

To further assess the influence of synbiotics on anxiety-like behaviors, behavioral tests evaluating spontaneous locomotor activity were conducted (Figure 1G–J). In the open field test, the EPEC-infected group traveled a significantly shorter distance in the central zone—and had a lower ratio of center to total distance—compared to the CON group (*p* < 0.01; Figure 1G). Similarly, in the elevated plus maze test, the EPEC group spent less time and covered less distance in the open arms, with correspondingly lower ratios relative to total movement (*p* < 0.01; Figure 1H). Treatment with synbiotics markedly alleviated these anxiety-like behaviors (*p* < 0.05; Figure 1G–J).

These results indicate that probiotics, prebiotics, and synbiotics are all effective in alleviating both diarrheal symptoms and anxiety-like behavior in a mouse model of EPEC-induced diarrhea.

### 3.3. Impact of Synbiotic Supplementation on Colonic Histopathological Changes and Inflammation in EPEC-Induced Infant Diarrhea

**Figure 2:**
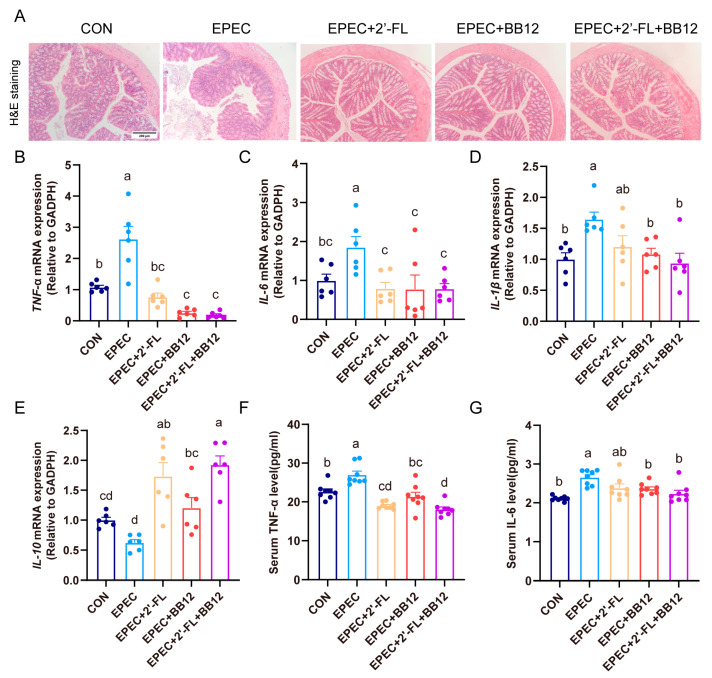
Impact of synbiotic supplementation on colonic histopathological changes and inflammation in EPEC-induced infant diarrhea. (**A**) colon H&E staining representative image; (**B**–**E**) Colon mRNA expression levels of TNF-α, IL-6, IL-1β, and IL-10 (*n* = 6); (**F**–**G**) serum levels of TNF-α and IL-6 (*n* = 8); Data are expressed as mean ± SEM. The letters (a–d) denote significant differences (*p* < 0.05) among the treatment groups.

H&E staining revealed that EPEC administration led to significant crypt distortion and reduction in goblet cells within the mucosal and submucosal layers of the colon. In contrast, treatment with synbiotics markedly alleviated these severe histological injuries (Figure 2A). Inflammatory responses in the colon and serum were further assessed. Relative to the control group, mice with EPEC-induced diarrhea showed a significant upregulation in the mRNA expression of pro-inflammatory cytokines in colonic tissue (Figure 2B–D). In contrast, the EPEC group exhibited a decrease in the mRNA level of IL-10—a key anti-inflammatory cytokine (Figure 2E). Notably, synbiotic treatment reduced the mRNA expression of TNF-α, IL-6, and IL-1β by 92.9%, 43.1%, and 57.7%, respectively, while increasing IL-10 expression by 208.7% (*p* < 0.01; Figure 2B–E). Additionally, synbiotic supplementation significantly lowered serum levels of TNF-α by 33% and IL-6 by 15.8% in the EPEC group (*p* < 0.01; Figure 2F,G). Taken together, these findings demonstrate that synbiotic intervention exerted a significant effect in alleviating colon histopathological injury and reducing inflammation at both local and systemic levels in mice suffering from EPEC-induced diarrhea.

### 3.4. Impact of Synbiotic Supplementation on Gut Barrier Integrity Damage in EPEC-Induced Infant Diarrhea

**Figure 3:**
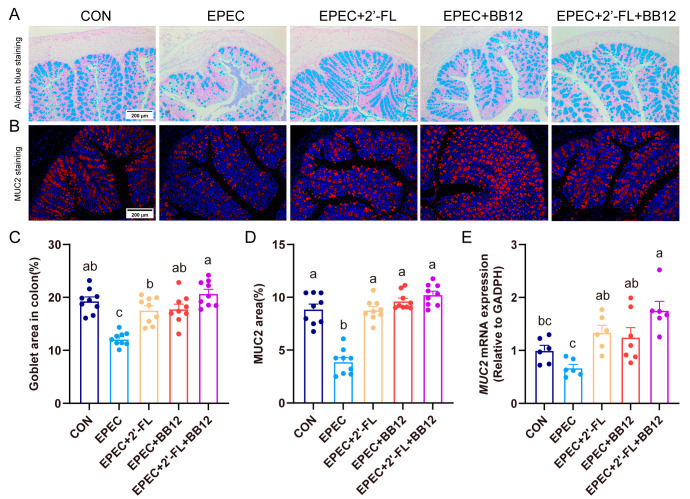
Impact of synbiotic supplementation on goblet cells function in EPEC-induced infant diarrhea. (**A**) colon Alcian blue staining representative images; (**B**) colon MUC2 immunofluorescence staining representative images; (**C**) goblet cell quantification based on Alcian blue staining with ImageJ software (*n* = 3); (**D**) MUC2 quantification based on immunofluorescence staining with ImageJ software (*n* = 3); (**E**) colon mRNA expression levels of MUC2 (*n* = 6). Data are expressed as mean ± SEM. The letters (a–c) denote significant differences (*p* < 0.05) among the treatment groups.

To investigate how synbiotics influence the damage to gut barrier integrity in EPEC-induced diarrhea mice, Alcian blue staining was utilized; additionally, this staining technique was applied to determine the count of goblet cells (Figure 3A). A valid decrease in colonic goblet cells was observed in EPEC-induced mice relative to controls (*p* < 0.01; Figure 3C). Conversely, synbiotic intervention significantly restored goblet cell numbers in the EPEC-induced group. (*p* < 0.01; Figure 3C). Synbiotics exhibited a significantly more potent intervention effect on mice with diarrhea compared to prebiotics (*p* < 0.05; Figure 3C). In addition, immunofluorescence staining was also used to evaluate the expression of MUC2, a mucin produced by goblet cells. Quantitative results revealed that synbiotic treatment markedly elevated colonic MUC2 expression by 163% in EPEC-induced mice (*p* < 0.01; Figure 3B,D). Moreover, MUC2 mRNA expression levels in the colon were also evaluated. Results showed that MUC2 mRNA expression levels in the colon increased by EPEC and were reduced by synbiotic treatment (*p* < 0.01; Figure 3E).

**Figure 4:**
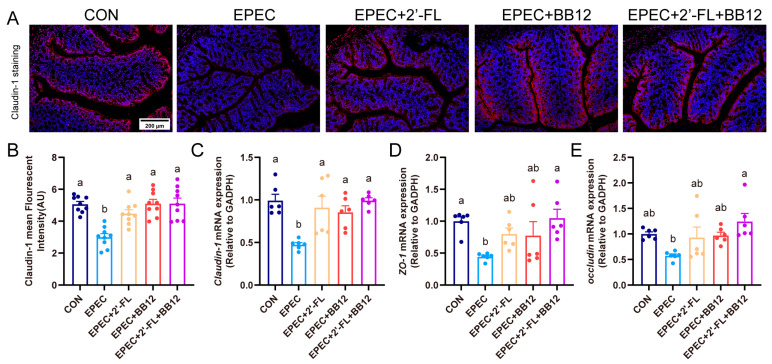
Impact of synbiotic supplementation on gut barrier tight-junction protein expressions in EPEC-induced diarrhea mice. (**A**) colon Claudin-1 immunofluorescence staining representative images; (**B**) claudin-1 quantitative analysis based on immunofluorescence staining with ImageJ software (*n* = 3); (**C**–**E**) colon mRNA expression levels of Claudin-1, ZO-1, and Occludin (*n* = 6). Data are expressed as mean ± SEM. The letters (a,b) denote significant differences (*p* < 0.05) among the treatment groups.

To assess colon barrier integrity, immunofluorescence staining was employed to evaluate claudin-1 expression. A notable decline in the average level of claudin-1 fluorescence intensity was observed in the colonic tissue of EPEC-induced mice relative to CON animals (*p* < 0.01; Figure 4A,B). However, a marked attenuation of this reduction was observed following treatment with prebiotic (49.5%), probiotic (70.4%), or synbiotic (70.3%) supplements (*p* < 0.01; Figure 4A,B). Moreover, consistent with the colon immunofluorescence staining results, RT-qPCR results suggested that the mRNA expression of three key proteins for tight junctions, Claudin-1, ZO-1, and Occludin, was significantly up-regulated by 108.9%, 136.1%, and 116.6% in the EPEC-induced group (Figure 4C–E). Together, these results indicated that synbiotics could mitigate EPEC-caused disruption of gut integrity and increased permeability.

### 3.5. Impact of Synbiotic Supplementation on Gut Microbiota Structure in Epec-Induced Infant Diarrhea

**Figure 5:**
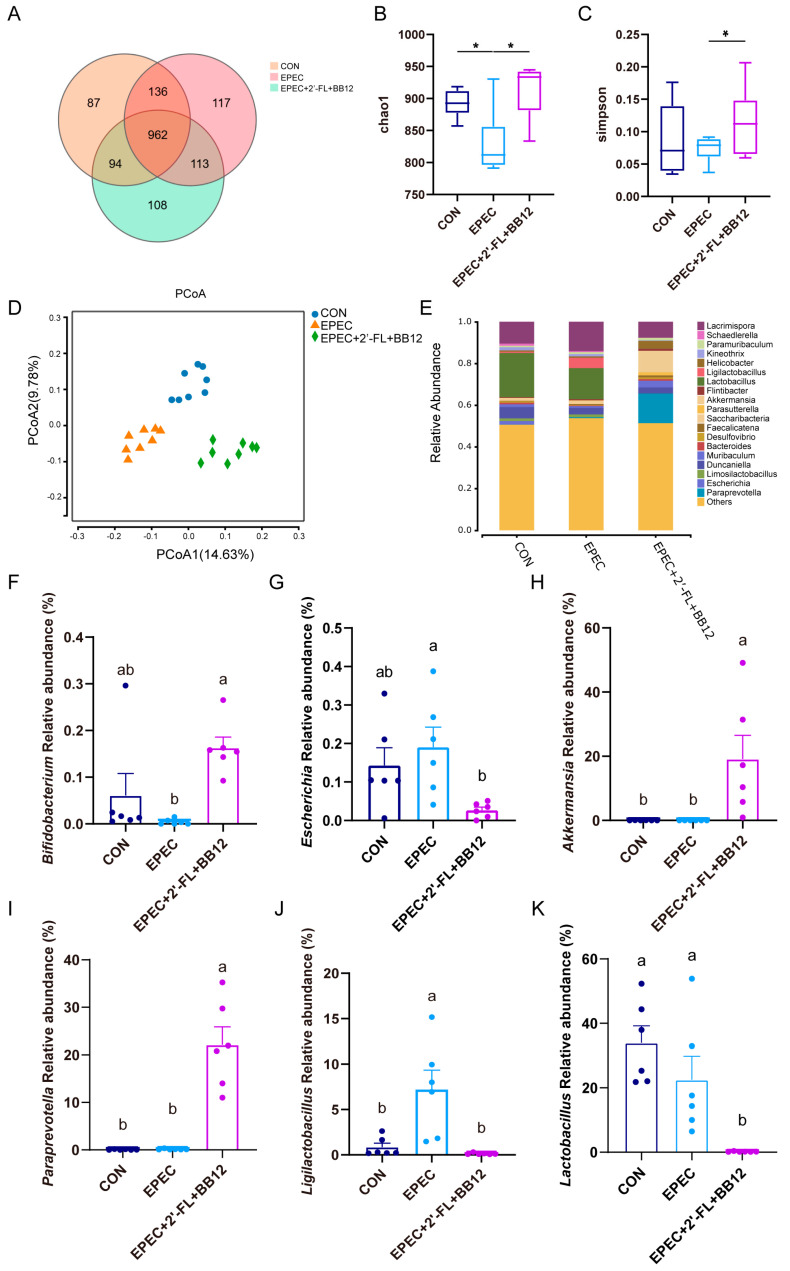
Impact of synbiotic supplementation on gut microbiota structure in EPEC-induced infant diarrhea. (**A**) Venn diagram of the various groups (*n* = 8); (**B**) Chao 1 index (*n* = 8); (**C**) Simpson 1 index (*n* = 8); (**D**) Principal Coordinate Analysis (PCoA) diagram of each group (*n* = 8); (**E**) The gut microbiota composition is presented at the genus level, with all genera exhibiting an average relative abundance below 1% grouped into the “other” category (*n* = 8); (**F**–**K**) relative abundance of *Bifidobacterium*, *Escherichia*, *Akkermansia*, *Paraprevotella*, *Ligilactobacillus*, and *Lactobacillus* (*n* = 6). Data presented as mean ± SEM. The letters (a,b) denote significant differences (*p* < 0.05) among the treatment groups. Statistical significance: * *p* < 0.05.

To assess how synbiotic treatment affects gut microbial diversity in diarrheal mice, fecal samples from the control group, EPEC-induced group, and EPEC + 2′-FL + BB12 group underwent 16S rRNA sequencing analysis (Figure 5). Venn analysis identified 87, 117, and 108 unique OTUs in the control, EPEC-induced, and synbiotic-treated groups, respectively (Figure 5A). Alpha diversity, assessed using the Chao1 and Simpson indices, indicated a significant reduction in microbial richness and diversity in the EPEC group compared to the control. Synbiotic supplementation was found to markedly restore these alpha diversity measures (Figure 5B,C). Principal coordinate analysis (PCoA) revealed apparent separation between each of the three groups, reflecting distinct microbial community structures. Specifically, the synbiotic intervention group formed a cluster separate from the EPEC group, indicating a structural shift in the microbiota composition (Figure 5D).

At the genus level, synbiotic administration notably elevated the abundance of Bifidobacterium by 37.3% (*p* < 0.05; Figure 5E,F), while reducing Escherichia by 81.2% (*p* < 0.05; Figure 5E,G). Furthermore, remarkable increases were observed in *Akkermansia* and *Paraprevotella*, along with significant reductions in *Ligilactobacillus* (98.3%) and *Lactobacillus* (99.2%), relative to the EPEC-induced group (*p* < 0.01; Figure 5H–K). These results demonstrate that synbiotic intervention effectively alleviates EPEC-induced gut microbiota dysbiosis.

### 3.6. Impact of Synbiotic Supplementation on Scfas Production and Their Colonic Receptors Expressions in Epec-Induced Infant Diarrhea

**Figure 6:**
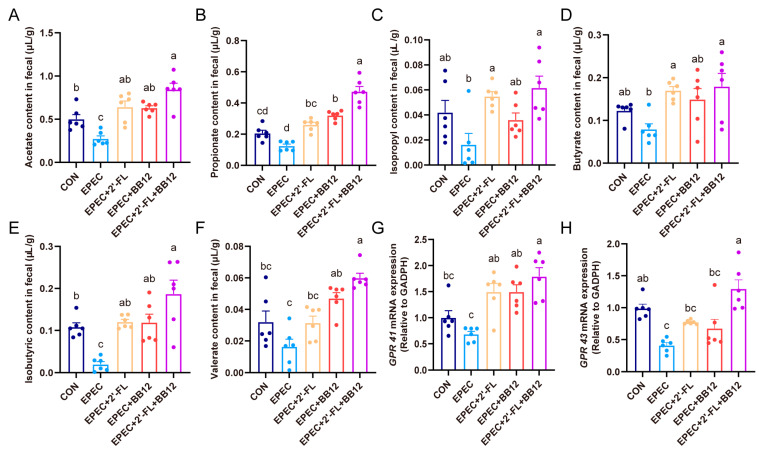
Impact of synbiotic supplementation on SCFAs production and their colonic receptors’ expressions in EPEC-induced infant diarrhea. (**A**–**F**) fecal concentrations of acetate, propionate, isobutyrate, butyrate, and valerate; (**G**,**H**) colon mRNA expression of GPR41 and GPR43. Data presented as mean ± SEM (*n* = 6). The letters (a–d) denote significant differences (*p* < 0.05) among the treatment groups.

To assess how synbiotic supplementation affects SCFAs production in diarrheal mice, the levels of fecal SCFAs were determined. In comparison with the control group, mice with EPEC-induced diarrhea showed a significant reduction in fecal acetate and isobutyrate contents (*p* < 0.05; Figure 6A,E). Synbiotic treatment notably increased the levels of SCFAs in EPEC-induced mice (*p* < 0.05; Figure 6A–F). Moreover, propionate and valerate levels in synbiotic-treated diarrheal mice were significantly higher than those receiving either probiotic or prebiotic interventions alone (*p* < 0.05; Figure 6B,F). Additionally, synbiotic administration significantly up-regulated the mRNA expression of SCFAs receptors GPR41 and GPR43 in EPEC-challenged mice (*p* < 0.05; Figure 6G,H). These findings suggest that synbiotic intervention effectively restored SCFAs production in EPEC-induced diarrheal mice.

### 3.7. Correlation Analysis Between Diarrhea Index, Inflammation, Gut Microbiota, and Other Biochemical Indexes in EPEC-Induced Infant Diarrhea

**Figure 7:**
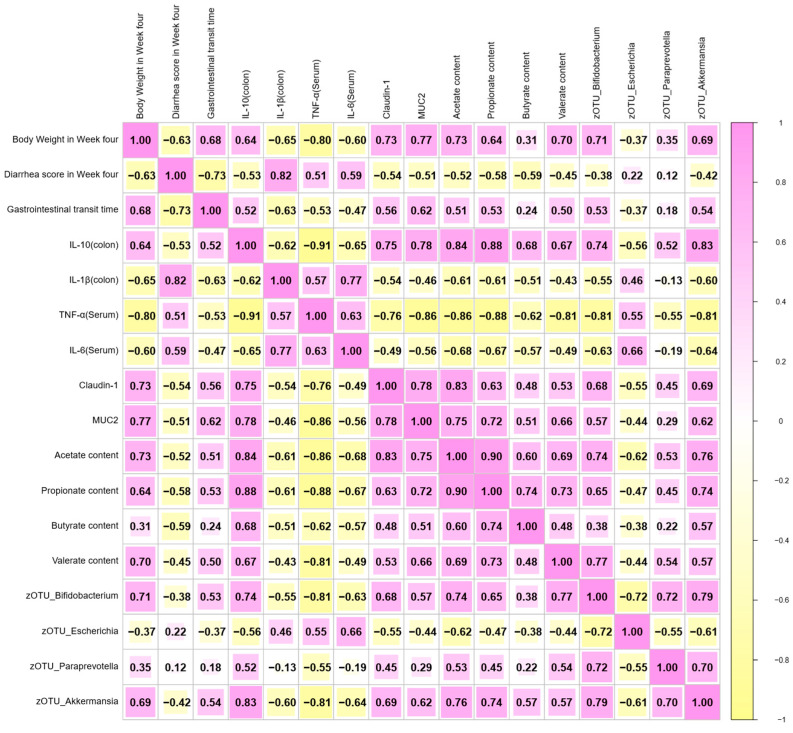
Spearman’s correlation analysis of diarrhea indices, inflammatory markers, gut microbiota, and biochemical parameters in an EPEC-induced infant diarrhea. Spearman’s correlation analysis was conducted using data from the CON, EPEC, and EPEC + 2′-FL + BB12 groups (*n* = 6). A legend in the upper right corner indicates the color and size of the circles, which represent the magnitude and direction of the correlations, with red denoting positive and blue denoting negative correlations. The corresponding numerical correlation values are shown in the lower left corner.

Correlation analyses were performed among diarrhea indices, gut microbiota composition, gut inflammation markers, and other biochemical parameters in young mice from the CON, EPEC, and EPEC + 2′-FL + BB12 groups (Figure 7). Spearman correlation analysis indicated that the inflammatory cytokines were positively correlated with diarrhea scores and negatively correlated with GITT. Conversely, levels of SCFAs demonstrated a positive correlative correlation with the abundance of *Bifidobacterium*, *Paraprevotella*, *Akkermansia*, and the anti-inflammatory cytokine IL-10, while being inversely correlated with pro-inflammatory factors. Both mucin MUC2 and tight junction protein Claudin-1 were positively associated with SCFAs levels. Furthermore, SCFA-promoting bacteria were negatively correlated with inflammatory responses. These results suggest a close relationship between synbiotic supplementation and the amelioration of EPEC-induced diarrhea, modulation of gut microbiota, reduction in inflammation, and promotion of SCFAs production.

## 4. Discussion

This study examined the impact of a synbiotic formulation containing 2′-FL as a carbon source on EPEC-induced diarrhea and gut barrier function in an infant model. The results demonstrated that co-administration of BB-12 and 2′-FL markedly alleviated diarrheal symptoms, as indicated by reduced diarrhea scores, prolonged GITT, and diminished anxiety-like behaviors. Furthermore, the synbiotic treatment restored gut morphology, attenuated inflammatory responses, enhanced gut barrier integrity, and modulated the gut microbiota composition. It also elevated fecal levels of SCFAs and up-regulated the expression of their corresponding receptors.

The results demonstrate that while both BB-12 and 2′-FL offer partial protection, their synbiotic combination provides superior efficacy in alleviating diarrhea and restoring GITT (Figure 1). In vitro experiments reveal that BB-12 exhibits remarkable metabolic efficiency in 2′-FL-enriched environments, as evidenced by its accelerated entry into the stationary growth phase when 2′-FL serves as the sole carbon source (Figure 1A). BB-12 efficiently utilizes 2′-FL, a trait not universal among probiotics [19]. The metabolic specialization not only drives BB-12 proliferation but also generates cross-feeding metabolites such as lactic acid, which facilitates the colonization of symbiotic partners like Lactobacillus through nutrient sharing [28]. Such microbial synergy establishes a mutually reinforcing ecological network that enhances gut microbiota resilience. Notably, the therapeutic potential of BB-12 may operate through dual mechanisms: competitive exclusion of enteropathogens like EPEC via niche occupation, and microenvironment modulation through acidification of the gut lumen. These findings provide mechanistic justification for pairing BB-12 with 2′-FL as synbiotic formulations. Furthermore, the alleviation of anxiety-like behaviors (Figure 1) provides strong support for the emerging concept of the microbiota–gut–brain axis in the context of infectious disease. The reduction in systemic inflammation and potential modulation of microbial metabolites by the synbiotic likely contribute to mitigating the neurobehavioral comorbidities of gut infection, a finding consistent with recent systematic reviews linking gut dysbiosis to anxiety disorders [6,29].

Previous studies have clearly demonstrated that EPEC infection can lead to gut tissue damage and inflammatory cascade reactions [30]. In this study, the colonic pathological characteristics of EPEC-infected young mice were further verified, including thinning of the muscle layer, destruction of crypt structures, upregulated expressions of pro-inflammatory cytokines, and decreased mRNA levels of the anti-inflammatory factor IL-10 (Figure 2). Notably, although earlier research has exhibited that Bifidobacterium animalis can alleviate gut inflammation and reverse gut barrier dysfunction [31,32], this study is the first to confirm that synbiotic intervention exhibits a more potent synergistic effect in improving gut tissue damage through simultaneous suppression of pro-inflammatory factors and promotion of anti-inflammatory factors (Figure 2). 2′-FL is capable of boosting the proliferation of intestinal epithelial cells and the regeneration of goblet cells, while also strengthening barrier integrity through the upregulation of the expressions of mucin MUC2 and tight junction protein Claudin-1 [33,34]. This study found that synbiotics specifically upregulated MUC2 expression and increased the number of goblet cells, suggesting that they may strengthen the mucus barrier through direct activation of goblet cells or indirect regulation by microbial metabolites such as SCFAs (Figure 3). Furthermore, synbiotic intervention enhanced the integrity of the colonic epithelial barrier, as demonstrated by upregulated Claudin-1 protein expression and increased mRNA levels of tight junction proteins including Claudin-1, MUC2, ZO-1, and Occludin (Figure 4).

Establishing a stable gut microbiota during infancy is crucial for immune system maturation, nutrient metabolism, and pathogen resistance [35]. Disruption of this fragile ecosystem, especially by gut pathogens such as EPEC, can lead to dysbiosis characterized by reduced microbial diversity and proliferation of harmful taxa, thereby increasing susceptibility to diarrhea [36]. A study has demonstrated that BB-12 modulates the gut microbiota by elevating the abundance of beneficial bacteria (e.g., the genus *Ligilactobacillus*) and reducing the relative abundance of harmful bacteria (e.g., *Escherichia*) [15]. Building on this, in this study, synbiotic intervention combining BB12 and 2′-FL significantly reduced the abundance of *Escherichia* while enriching beneficial genera such as *Bifidobacterium*, *Akkermansia*, and *Paraprevotella* (Figure 5). *Akkermansia* and *Paraprevotella* can produce SCFAs such as acetate, and this metabolic activity likely inhibits pathogen survival by reducing the gut pH [37]. *Akkermansia*, in particular, is a keystone species that resides in the mucus layer and is known to directly cross-talk with gut epithelial cells to enhance mucus thickness and barrier function [38]. Notably, different from previous studies, the abundances of *Lactobacillus* and *Ligilactobacillus* in the feces of mice in the synbiotic intervention group also decreased significantly. This observation could potentially be associated with competitive relationships with *Akkermansia* and *Paraprevotella*—microbes that occupied a large ecological niche in the gut environment—though other unaccounted factors cannot be completely excluded. This observation suggests that synbiotics have the ability to restructure the spatial distribution of microbiota through modulating interspecies interactions—for instance, competition for nutrients or the inhibition of quorum sensing.

Evidence indicates that the organic acids synthesized through probiotic metabolism of HMOs contribute to an acidic milieu with antibacterial properties [39]. As key metabolites of gut microbiota, SCFAs regulate cytokine secretion through the MAPK signaling pathway by activating G protein-coupled receptors (GPR41/GPR43) on gut epithelial cells, thereby mediating tissue inflammatory responses [40]. In this study, synbiotic intervention significantly increased the levels of SCFAs and their receptors in the intestines of EPEC-infected young mice. In addition, SCFAs may enhance gut barrier function by increasing tight junction proteins, potentially through the AKT/mTOR pathway [35]. This mechanism, however, has not been experimentally confirmed in the current model. Therefore, the enrichment of *Akkermansia* and *Paraprevotella* may be a key driving factor for the increase in SCFAs levels, and synbiotics may regulate the diarrhea process by upregulating the abundance of acid-producing bacteria.

In conclusion, synbiotic supplementation may reduce gut inflammation by enhancing the abundance of SCFA-producing bacteria and elevating SCFA levels, which suppresses pro-inflammatory cytokines and stimulates anti-inflammatory IL-10 release. Concurrent upregulation of Claudin-1 and MUC2 expression further reinforces gut barrier integrity. While this study highlights the pivotal role of SCFAs, the receptor-mediated signaling pathways require further systematic investigation. Future studies employing approaches such as metabolite receptor gene knockout models may help elucidate the underlying mechanisms. Moreover, based on current animal evidence, clinical trials are warranted to assess the safety and efficacy of synbiotics in diarrheal infants, and to evaluate the preventive and therapeutic potential of HMOs combined with probiotics.

## 5. Conclusions

In conclusion, the synbiotic combination of BB12 and 2′-FL markedly ameliorated diarrhea and anxiety-like behaviors induced by EPEC. It also modulated colonic inflammation and promoted the restoration of the gut barrier, mechanisms potentially mediated through the modulation of gut microbiota—such as *Akkermansia* and *Paraprevotella*—and increased generation of microbial metabolites, particularly SCFAs. These results, based on a mouse model of EPEC infection, support the potential of this synbiotic as a candidate nutritional strategy for addressing diarrhea in a preclinical setting, and underscore the therapeutic promise of BB12 and 2′-FL supplementation. However, given the inherent differences between murine and human gut physiology and microbiota, further validation through well-designed human clinical trials is essential before any inference of applicability to infantile diarrhea can be made.

## Data Availability

The raw data supporting the conclusions of this article will be made available by the authors on request.

## Abbreviations

The following abbreviations are used in this manuscript:

HMOs	human milk oligosaccharides
EPEC	enteropathogenic *Escherichia coli*
*E. coli*	*Escherichia coli*
2′-FL	2′-fucosyllactose
SCFAs	short-chain fatty acids
GITT	Gastrointestinal transit time
PBS	phosphate-buffered saline
LB	Luria–Bertani
H&E	Hematoxylin and eosin
PCA	principal component analysis
OTU	operational taxonomic units

## Appendix A

**Table A1:** Primer sequences used for semi-quantitative RT-PCR analysis.

Primer	Forward 5′-3′	Reverse 5′-3′
GAPDH	TGGAGAAACCTGCCAAGTATGA	TGGAAGAATGGGAGTTGCTGT
TNF-α	CTCATGCACCACCATCAAGG	ACCTGACCACTCTCCCTTTG
IL-1β	AGCTGGAGAGTGTGGATCCC	CCTGTCTTGGCCGAGGACTA
IL-6	CCACTTCACAAGTCGGAGGC	GGAGAGCATTGGAAATTGGGGT
IL-10	GCTCCAAGACCAAGGTGTCTACAA	CCGTTAGCTAAGATCCCTGGATCA
ZO-1	TGGGAACAGCACACAGTGAC	GCTGGCCCTCCTTTTAACAC
Claudin-1	AGCTGCCTGTTCCATGTACT	CTCCCATTTGTCTGCTGCTC
MUC2	GCTGACGAGTGGTTGGTGAATG	GATGAGGTGGCAGACAGGAGAC
occludin	TGGCTATGGAGGCGGCTATGG	AAGGAAGCGATGAAGCAGAAGGC
GPR41	GTGACCATGGGGACAAGCTTC	CCCTGGCTGTAGGTTGCATT
GPR43	GGCTCCCTGCCAACCTGCTG	GTGCACAGGGGCAGGCTGAG
